# Supplementary material for: Dual-gate organic phototransistor with high-gain and linear photoresponse
Source: Nat Commun. 2018 Oct 31;9:4546. doi: 10.1038/s41467-018-06907-6 (PMC6208338; doi:10.1038/s41467-018-06907-6)
Supplement: Supplementary file 1 — Supplementary Information [file 41467_2018_6907_MOESM1_ESM.pdf]

Dual-gate organic phototransistor with high-gain and linear photoresponse

Authors: Philip C.Y. Chow[1†*], Naoji Matsuhisa[1], Peter Zalar[1,2‡], Mari Koizumi[1,2], Tomoyuki Yokota[1,2], Takao Someya[1,2*]

Affiliations

[1]Department of Electrical Engineering and Information Systems, Graduate School of Engineering, The University of Tokyo, Japan

[2]Exploratory Research for Advanced Technology (ERATO), Japan Science and Technology Agency (JST), 2-11-16, Yayoi, Bunkyo-ku, Tokyo, 113-0032, Japan.

[†]Present address: Department of Chemistry, The Hong Kong University of Science and Technology, Clear Water Bay, Kowloon, Hong Kong

[‡]Present address: Holst Centre/TNO, High Tech Campus 31, 5656 AE, Eindhoven, The Netherlands

[*]Correspondence to: P.C.Y.C <pcyc@ust.hk>, T.S. <someya@ee.t.u-tokyo.ac.jp>

Supplementary Figures 1 - 13

Supplementary Table 1

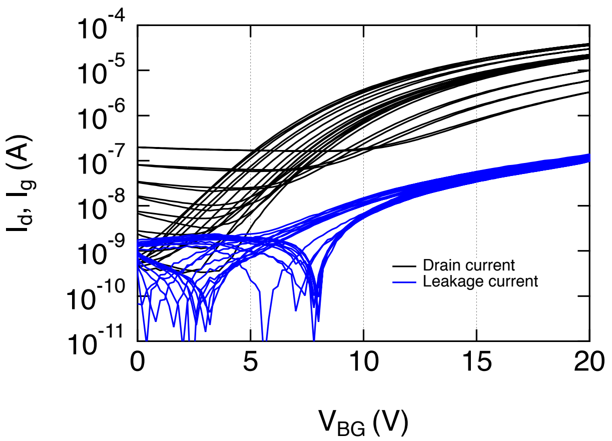

Supplementary Figure 1. Transfer characteristics of dual-gate MDMO-PPV:PCBM (1:15 blend ratio by weight) phototransistor showing leakage current through gate electrodes (I_g) and drain current (I_d) at a range of top gate biases. The relatively low leakage current compared to the drain current, along with the lack of hysteresis observed, indicates that the gate dielectrics are not damaged under these operation bias conditions.

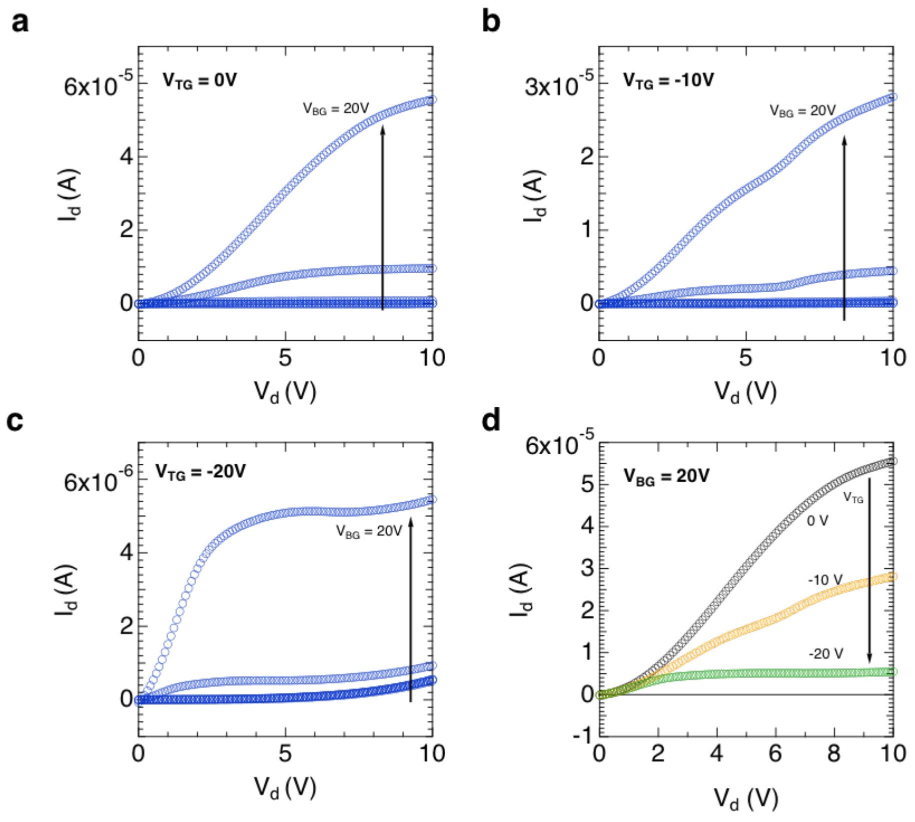

Supplementary Figure 2. Output characteristics of dual-gate MDMO-PPV:PCBM phototransistor showing drain current (I_d) as a function of source-drain bias (V_d) with increasingly positive bottom gate bias (V_{BG}) and fixed top gate bias V_{TG} = 0V (panel a), V_{TG} = -10 V (panel b), and V_{TG} = -20 V (panel c). At V_{TG} = -20 V, the output curves clearly indicate ambipolar transport. Panel d summaries the decrease of I_d by increasingly negative V_{TG}, with V_{BG} fixed at 20 V.

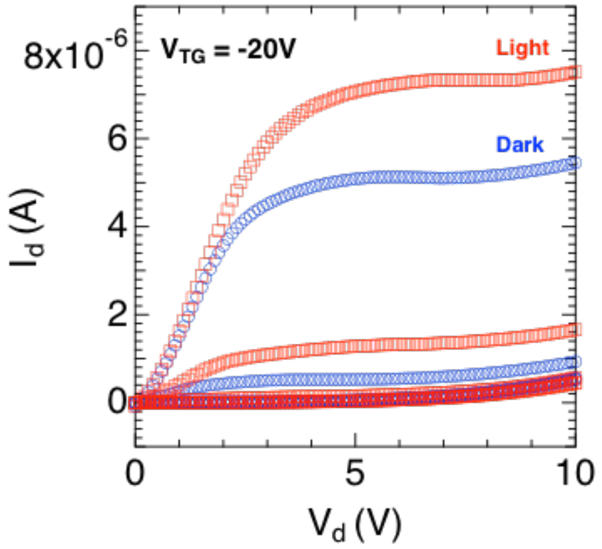

Supplementary Figure 3. Output characteristics of dual-gate MDMO-PPV:PCBM phototransistor at V_{TG} = -20 V and V_{BG} = 20 V, showing increase in drain current (I_d) upon light illumination (0.5 mW cm^{-2} at 540 nm).

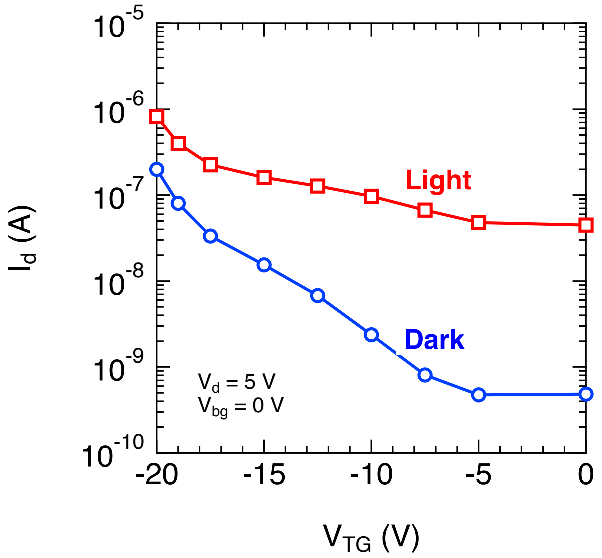

Supplementary Figure 4. Transfer characteristics of dual-gate MDMO-PPV:PCBM phototransistor with increasing negative V_{TG}, with constant V_{BG} = 0 V and V_d = 5 V. The increase in channel current due to negative V_{TG} is analogous to that shown in Fig. 2a and Fig. 2c. Photoresponse is created upon light illumination (45 mW cm^{-2} at 540 nm).

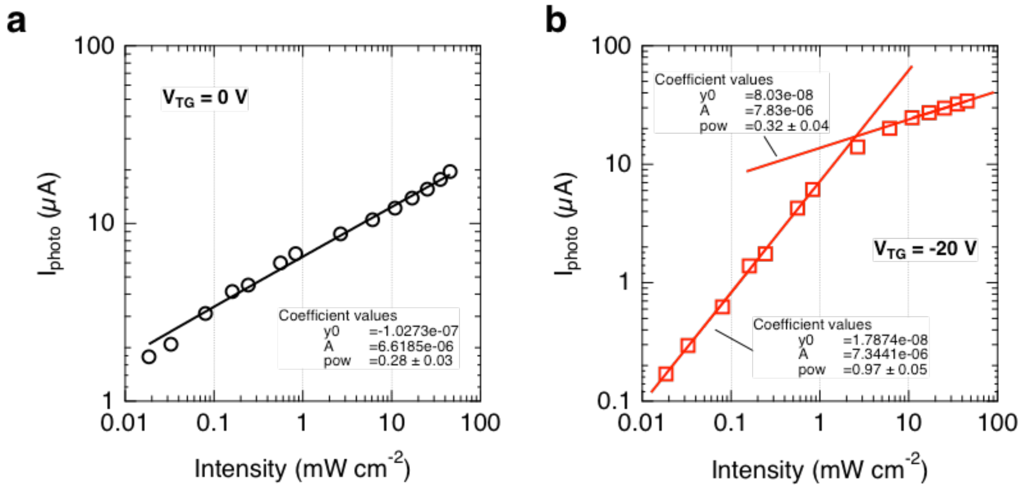

Supplementary Figure 5. Light intensity dependence of photocurrent (I_{photo}, defined as the drain current in light subtracted by that in the dark). The data was fitted using a power law: $y = y_0 + Ax^{pow}$. Without applying top gate bias (panel a), photocurrent scales sublinearly with increasing intensity throughout the studied range. Such sublinear photoresponse is typically found in conventional phototransistors. When operating with a negative top gate bias (panel b), the photoresponse scales linearly (sublinearly) with intensity below (above) 2 mW cm^{-2}.

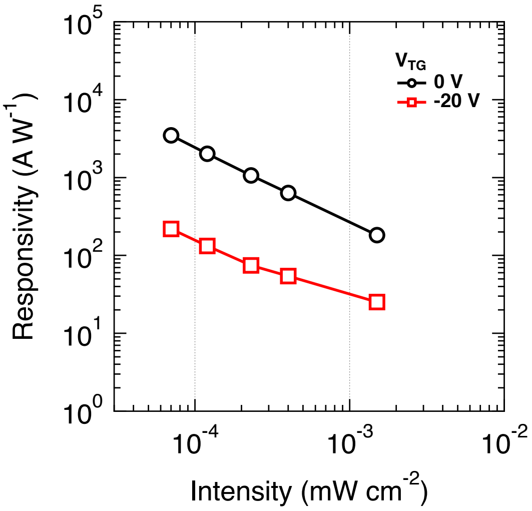

Supplementary Figure 6. Intensity dependence of device photo-responsivity at very low irradiance. Device exhibits sublinear response at both bias conditions in this range. At such low irradiance, it is likely that carriers are required to fill up deep lying trap states before they can be transported to the electrodes. These deep lying traps contribute to photoconductive gain in both operation modes.

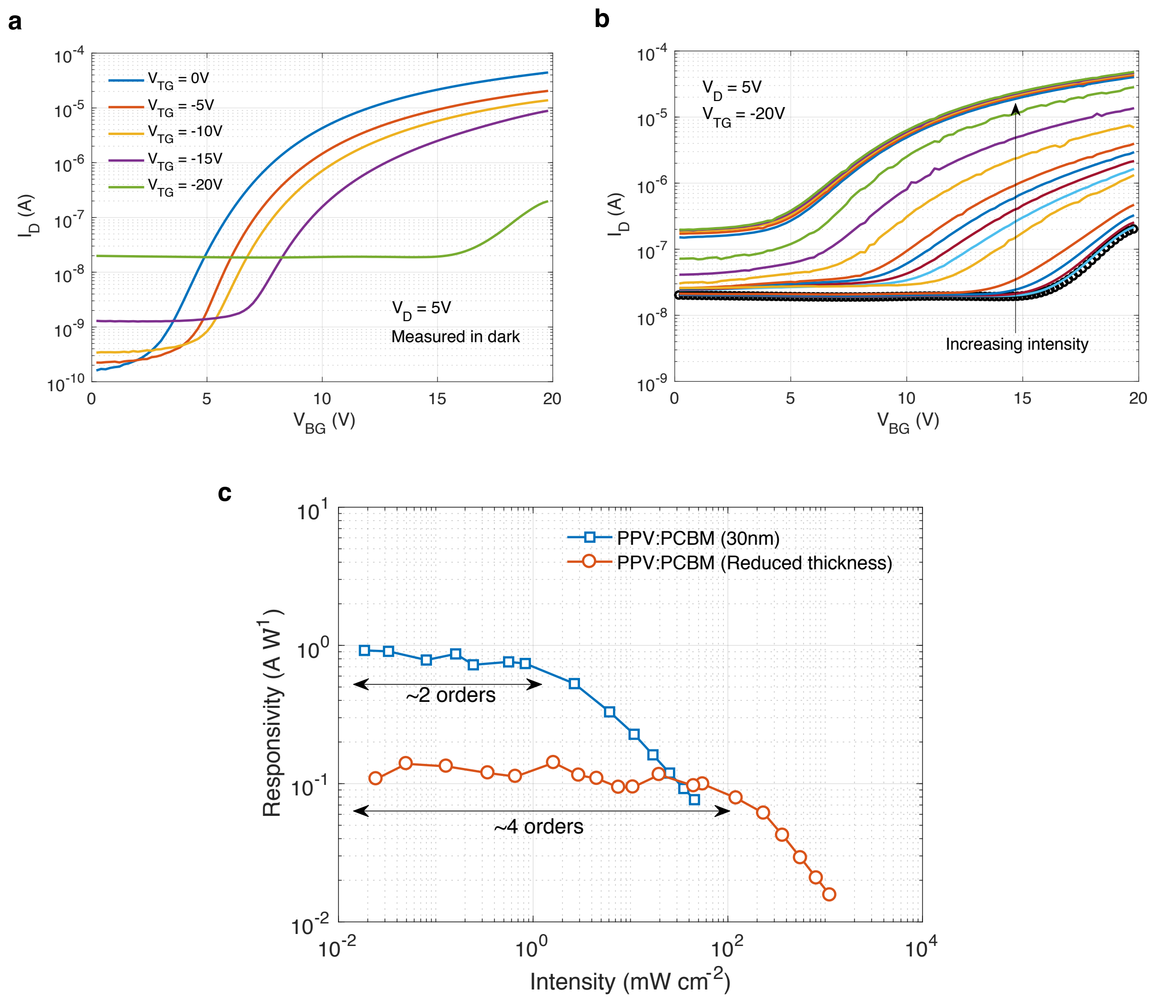

Supplementary Figure 7. Increased linear dynamic range of MDMO-PPV:PCBM dual-gate phototransistor with reduced thickness. This causes a larger threshold voltage shift than in the original device, causing larger channel depletion and improves the linear dynamic range up to ~100 mW cm^{-2} (~4 orders) This result shows that the sub-linearity at high intensity (>1 mW cm^{-2}) is due to saturation of the bottom channel, and the linear range can be extended by further depleting this channel with the top gate bias. However, further channel depletion reduces the gain, leading to reduced responsivity.

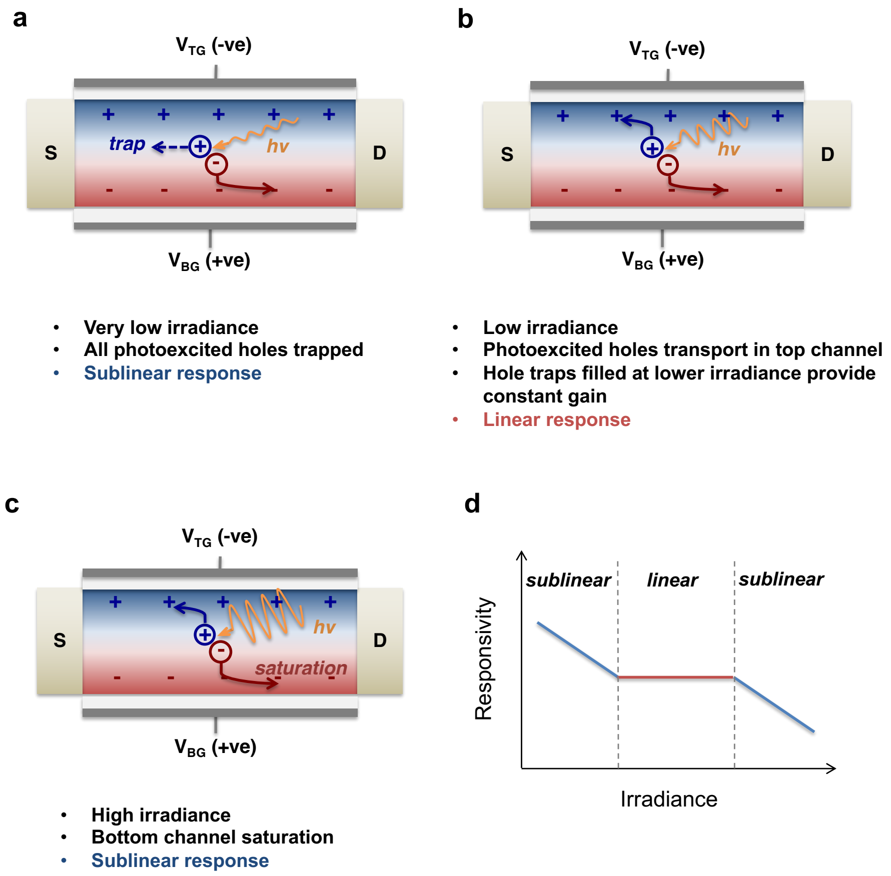

Supplementary Figure 8. Schematic illustration depicting the operation mechanism of dual-gate phototransistor at various irradiance conditions. In the interest of clarity, the schematic depicts the operation mechanism for an dual-gate device in which electrons are more mobile than holes, as in the case of MDMO-PPV:PCBM (with 1:15 blend ratio), but both n and p-type accumulation channels are present when the device is operating with opposite dual-gate bias (albeit with imbalanced mobility). At very low irradiance (panel a), all the photoexcited holes fill up trap sites, preferentially filling long-lived, energetically deep traps first. Additional holes fill up more shallow traps with decreasing lifetime that lead to small gain, therefore the device exhibits sublinear response as in the case of a unipolar phototransistor. At higher irradiance (panel b), the additional holes generated are transported across the device through the top channel created by the top gate bias, and do not affect the trapping density. In this case the trapping lifetime is unaffected by additional photoexcitations, and thus the device exhibits linear response (with constant gain provided by the traps filled up at lower irradiance). At even higher irradiance (panel c), the bottom channel reaches saturation, resulting in sublinear response. Panel d summarizes the relationship between response linearity and irradiance. In this study we employed the bottom gate to enable transport of the dominant carriers (electrons in the case of MDMO-PPV:PCBM at 1:15 ratio), and the top gate to modulate hole trapping density. We note that, in principle, the gates can be exchanged such that the top gate can drive the n-type transport (operating at positive bias), while the bottom gate can modulate hole trapping (operating at negative bias). In practice, however, the carrier mobility (both n and p type) may vary considerably when the gates are exchanged due to differences in interface roughness and morphology (Ref. 2), which in turn affect photo-responsivity and detectivity.

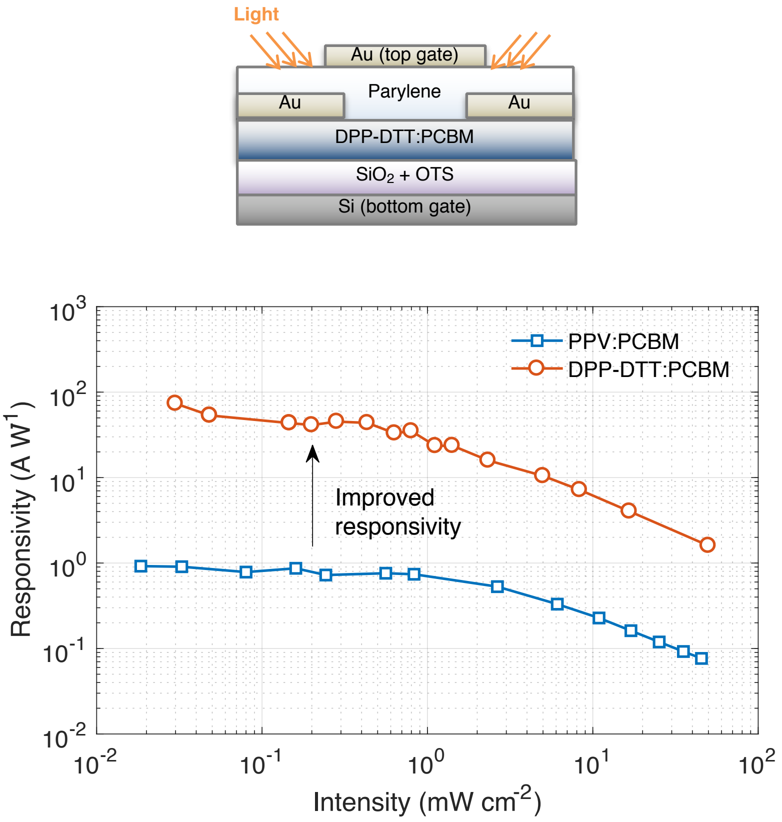

Supplementary Figure 9. Characterisation of dual-gate organic phototransistor based on DPP-DTT:PCBM. The source and drain electrodes were defined using a metal mask during thermal evaporation, with width and length of 700 µm and 30 µm, respectively. Despite operating mainly in the p-type regime (negative V_{BG} and positive V_{TG}), the optoelectrical behaviour of this device matches with the n-type MDMO-PPV:PCBM device (which operates with positive V_{BG} and negative V_{TG}). We observe linearization of the photoresponse below ~1 mW cm^{-2}, but with an improved responsivity up to ~40 A/W (EQE of ~9000%). We note that 40 A/W is a lower bound value because the device was photoexcited at 532nm (absorption peak of DPP-DTT is at 800nm) and the non-transparent top gate (30nm of gold) is likely to have blocked some of the incoming light. This result confirms that the proposed device concept is applicable to high-performance semiconducting systems, and much improved photo-responsivity can be achieved.

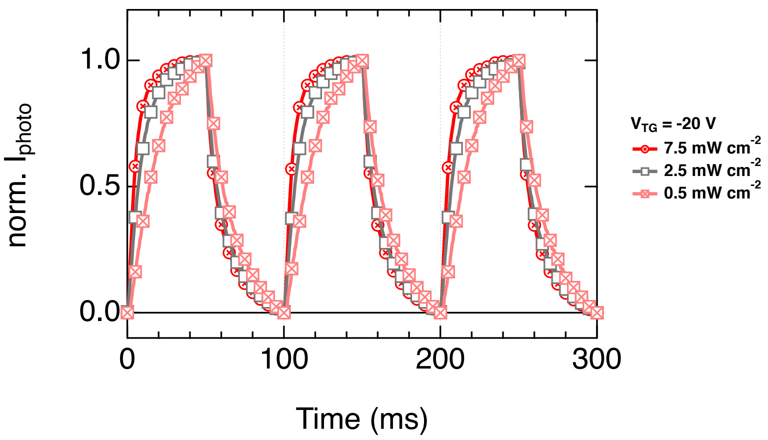

	7.5 mW cm^{-2}		0.5 mW cm^{-2}	
Top gate bias	0 V	-20 V	0 V	-20 V
Rise time	8 ms	15 ms	23 ms	32 ms
Decay time	34 ms	27 ms	37 ms	32 ms

Supplementary Figure 10. Transient photocurrent of dual-gate organic phototransistor operating at negative top gate bias of -20 V, illuminated with a light source modulated at 10 Hz using an optical chopper at different intensities. It is worth mentioning that there is no sign of persistent photoconductance (PPC), which is a common problem for phototransistors that limits the recovery of its drain current back to its initial level before illumination. Possible causes of PPC include charge trapping and bias stressing at interface. For instance, previous work showed that MDMO-PPV:PCBM single-gate phototransistor with poly-vinyl-alcohol (PVA) or divinyltetramethyldisiloxane-bis(benzocyclobutene) (BCB) as bottom dielectric layer suffers from PPC and do not recover to its initial state unless the device is thermally treated. The absence of undesirable PPC effect and current hysteresis of the device discussed herein suggests that parylene forms a trap-free interface with the selected BHJ blend.

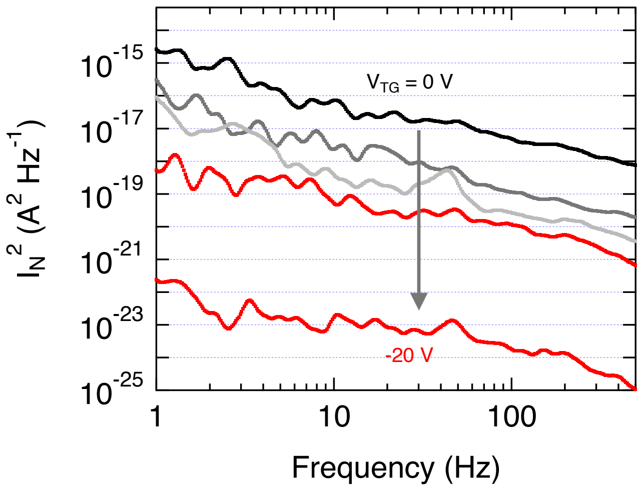

Supplementary Figure 11. Noise power spectral density of dual-gate organic phototransistor operating at a range of top gate biases, with bottom gate bias and source-drain bias fixed at 20 V and 5 V, respectively. The noise power spectra follows 1/f characteristics at all biases, which reflects that noise at this low frequency range is mainly due to 1/f (flicker) noise. The 1/f noise is often expressed by the empirical relation ${I_N}^2 = \frac{A{I_d}^{\alpha}}{f^{\beta}}$. The bias exponent α is ranged between 1-2 for organic transistors since carrier transport is two-dimensional in nature with surface trapping acting as the primary source of noise. By fitting our data with the above equation, we find that the noise coefficient A, which is a measure of the total number of active traps responsible for causing noise, is largely reduced by the top gate bias. Our calculated noise coefficient *A* drops from 9.8 x 10^{-10} A μm^2 to 2.7 x 10^{-15} A μm^2 as V_{TG} increased from 0 to -20 V when assuming α = 1.

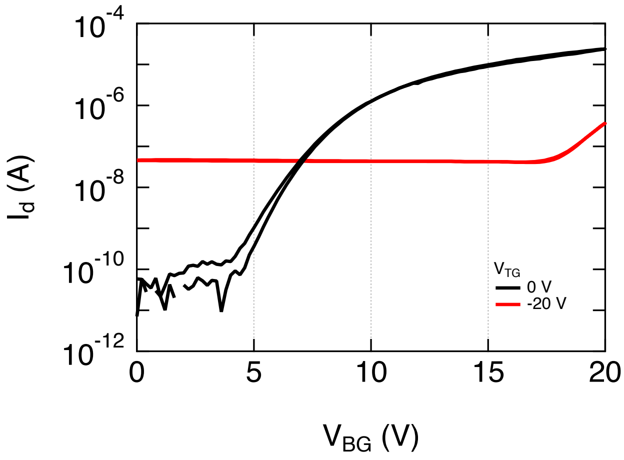

Supplementary Figure 12. Transfer curve of device in dark. Shot noise spectral density is given by $I_{shot} = \sqrt{2qI_{dark}}$. The shot noise spectral density is thus 2.7 x 10^{-12} A $Hz^{-1/2}$ and 3.1 x 10^{-13} A $Hz^{-1/2}$ for V_{TG} = 0 and -20 V, respectively. Assuming that the shot noise is the dominant source of noise in these devices, the specific detectivity values with and without applying negative V_{TG} are 3.7 x 10^{10} Jones (V_{TG} = 0 V) and 3.2 x 10^{11} Jones (V_{TG} = -20 V). These are at least an order of magnitude larger than the values calculated using the full noise power spectral density shown in Fig. S11.

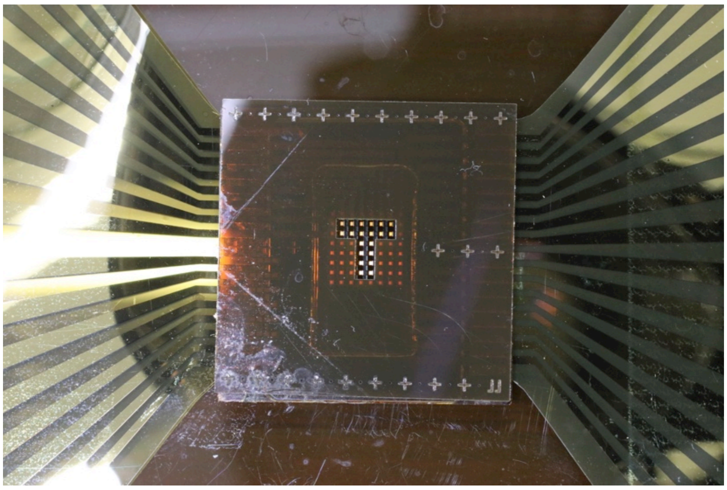

Supplementary Figure 13. Supporting image for image sensor demonstration. Since each column (8 pixels) share the same top and bottom gates, it was necessary to limit the cross-talk between these pixels by reducing V_{TG} from -20 V (used for single pixel charactierzation) to -17.5 V in the matrix arrangement.

Material	Detectivity		Reference
	1 mW cm^{-2}	100 nW cm^{-2}	
Silicon diode	10^{12} Jones	10^{12} Jones	
DPP-DPP:PCBM	1.1 x 10^{9} Jones	2 x 10^{13} Jones	Ref. 3
PBDTT-DPP:PCBM/IGZO	1.1 x 10^{9} Jones	3.9 x 10^{12} Jones	Ref. 4
PBDTT-DPP:PC71BM/ZnON	8.8 x 10^{10} Jones	8.8 x 10^{13} Jones	Ref. 5
MDMO-PPV:PCBM (Single-gated)	10^{7} Jones	10^{10} Jones	Ref. 1 & this work
**MDMO-PPV:PCBM (Dual-gated)**	**1.5 x 10^{10} Jones**	**1.5 x 10^{12} Jones**	**This work**

Supplementary Table 1. Comparison of the detectivity values of our proof-of-concept device (operating at single/dual gate mode) with those achieved in other state-of-the-art organic phototransistors and in silicon diode. Phototransistors exhibit sublinear response, and therefore higher detectivity values are achieved at reducing irradiance. In this table, the sublinear detectivity with respect to irradiance is colored in red, while the linear detectivity is colored in green.

Supplementary References

1. Anthopoulos, T. D., Electro-optical circuits based on light-sensing ambipolar organic field-effect transistors. *Appl. Phys. Lett.* 91, 113513 (2007).

2. Brondijk, J. J., Spijkman, M., Torricelli, F., Blom, P. W. M. & de Leeuw, D. M., Charge transport in dual-gate organic field-effect transistors. *Appl. Phys. Lett.* 100, 023308 (2012).

3. Xu, H., Liu, J., Zhang, J., Zhou, G., Luo, N. & Zhao, N., Flexible organic/inorganic hybrid near-infrared photoplethysmogram sensor for cardiovascular monitoring, *Adv. Mater.* 1700975 (2017).

4. Rim, Y. S. et al., Ultrahigh and Broad Spectral Photodetectivity of an Organic–Inorganic Hybrid Phototransistor for Flexible Electronics. Adv. Mater. 27, 6885-6891 (2015).

5. Rim, Y. S. et al., Boosting Responsivity of Organic–Metal Oxynitride Hybrid Heterointerface Phototransistor, *ACS Appl. Mater. Interfaces* 8 (23), 14665-14670 (2016)
